# Performance of the CYBERLEGs motorized lower limb prosthetic device during simulated daily activities

**DOI:** 10.1017/wtc.2021.15

**Published:** 2021-11-22

**Authors:** Jo Ghillebert, Joost Geeroms, Louis Flynn, Sander De Bock, Renée Govaerts, Elke Lathouwers, Simona Crea, Nicola Vitiello, Dirk Lefeber, Romain Meeusen, Kevin De Pauw

**Affiliations:** 1 Human Physiology and Sports Physiotherapy Research Group, Vrije Universiteit Brussel, Brussels, Belgium; 2 Brussels Human Robotic Research Center (BruBotics), Vrije Universiteit Brussel, Brussels, Belgium; 3 Department of Mechanical Engineering, Faculty of Applied Sciences, Vrije Universiteit Brussel and Flanders Make, Brussels, Belgium; 4 The BioRobotics Institute, Scuola Superiore Sant’Anna, Pisa, Italy; 5 Department of Excellence in Robotics & AI, Piazza Martiri della Libertà, Pisa, Italy; 6 Strategic Research Program ‘Exercise and the Brain in Health and Disease: The Added Value of Human-Centered Robotics’, Vrije Universiteit Brussel, Brussels, Belgium

**Keywords:** biomechanics, performance evaluation, physiology, prosthetics

## Abstract

**Background:**

The CYBERLEGs-gamma (CLs-ɣ) prosthesis has been developed to investigate the possibilities of powerful active prosthetics in restoring human gait capabilities after lower limb amputation.

**Objective:**

The objective of this study was to determine the performance of the CLs-ɣ prosthesis during simulated daily activities.

**Methods:**

Eight participants with a transfemoral amputation (age: 55 ± 15 years, *K*-level 3, registered under: NCT03376919) performed a familiarization session, an experimental session with their current prosthesis, three training sessions with the CLs-ɣ prosthesis and another experimental session with the CLs-ɣ prosthesis. Participants completed a stair-climbing-test, a timed-up-and-go-test, a sit-to stand-test, a 2-min dual-task and a 6-min treadmill walk test.

**Results:**

Comparisons between the two experimental sessions showed that stride length significantly increased during walking with the CLs-ɣ prosthesis (*p* = .012) due to a greater step length of the amputated leg (*p* = .035). Although a training period with the prototype was included, preferred walking speed was significantly slower (*p* = .018), the metabolic cost of transport was significantly higher (*p* = .028) and reaction times significantly worsened (*p* = .012) when walking with the CLs-ɣ compared to the current prosthesis.

**Conclusions:**

It can be stated that a higher physical and cognitive effort were required when wearing the CLs-ɣ prosthesis. Positive outcomes were observed regarding stride length and stair ambulation. Future prosthetics development should minimize the weight of the device and integrate customized control systems. A recommendation for future research is to include several shorter training periods or a prolonged adaptation period.

## Introduction

Performing daily activities is challenging for individuals with a transfemoral amputation. It is acknowledged that they have a higher metabolic cost and reduced physical performance during level and slope walking, stair climbing and rising from a chair compared to able-bodied individuals (Highsmith et al., 2011; Lura et al., 2015; Ledoux et al., 2017; Esposito et al., 2018). Movement patterns also differ compared to able-bodied individuals, for example, people with a transfemoral amputation often ambulate stairs step-by-step, whereas step-over-step is common for able-bodied individuals (Ledoux et al., 2017). Furthermore, an asymmetrical walking pattern is observed, and balance and stability are disturbed due to the lack proprioceptive information and loss of motor control (Williams et al., 2006; Eberly et al., 2014). These daily challenges and movement adaptations lead to the development of secondary complications such as lower back problems and discomfort of other joints (Wurdeman et al., 2018).

Common passive and quasi-passive lower limb prosthetic devices can already restore human functioning to a certain degree. However, the ultimate goal of prosthetic development is to restore at least part of daily participation so that the quality of life of individuals with a transfemoral amputation increases. Recent technological breakthroughs have evolved toward motorized prostheses (Martinez-Villalpando et al., 2011; Simon et al., 2013, 2014; Takahashi et al., 2015; Wu et al., 2017). Although some devices (e.g., the Power Knee from Össur, Reykjavik, Iceland) already reached the market, they are still under-prescribed and under-utilized due to the large costs of product development and customization (Lechler et al., 2018). Moreover, current motorized prostheses mainly focus on improving walking abilities, whereas emphasis on other daily activities is also of highly importance (Ghillebert et al., 2019).

**Figure 1.**
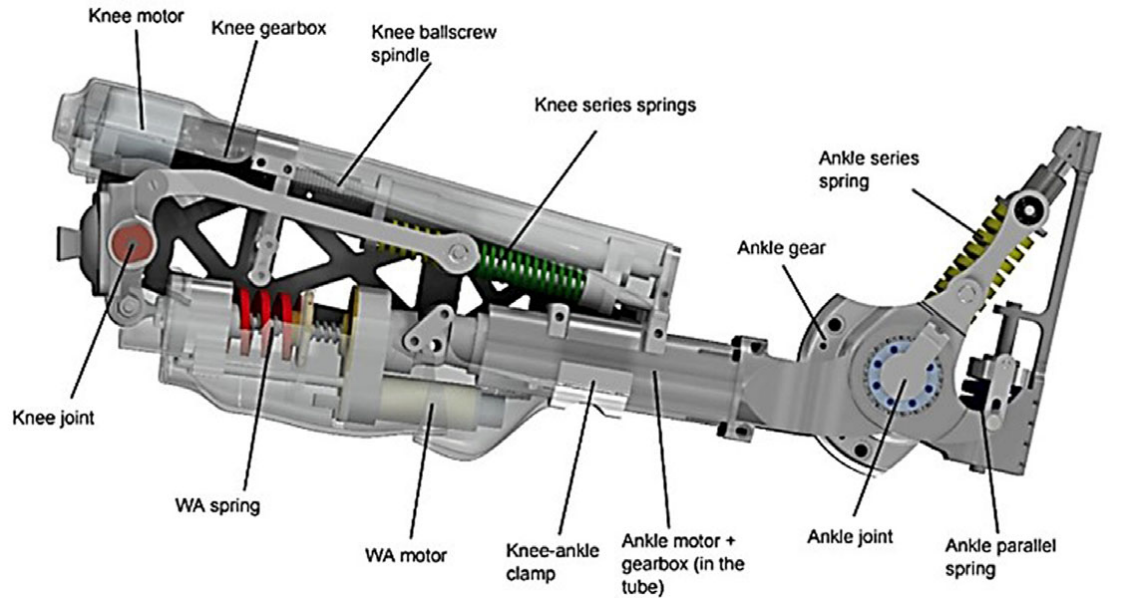
The CYBERLEGs-gamma prosthesis. The knee actuator consists of the motor, gearbox, and spindle, and the springs in series, acting on the knee joint through metal beams. The wearable apparatus consists of a motor moving the spring in and out of place. The ankle actuator consists of the motor, gearbox, and series and parallel springs acting on the ankle joint. The ankle and knee are clamped together, allowing a change in distance between the joints.

The CYBERLEGs-gamma (CLs-γ) prosthesis (Figure 1), the successor of the beta-version, has recently been designed and constructed by engineers from The CLs-γ prosthesis (Figure 1), the successor of the beta-version, has recently been designed and constructed by engineers from Vrije Universiteit Brussel (Flynn et al., 2018). Conceptually the prosthesis is similar to the beta-version, but more power was added to better assist individuals with a transfemoral amputation during more demanding tasks and adaptations have been made for improving robustness that allow for longer and more frequent test sessions. These improvements enabled to perform several simulated daily activities that were not investigated with the previous prototype (Flynn et al., 2015; Parri et al., 2017).

At the ankle joint, an actuator provides torque by compressing series elastic springs, whereas a parallel spring system acts between the shank and the foot to reduce energy consumption by storing potential and kinetic energy during dorsiflexion and releasing it during plantarflexion (Geeroms et al., 2017). At the knee joint, the actuator provides resistance also through a series elastic actuator, similar to eccentric muscle power, during the weight acceptance phase and is activated right before heel strike. Afterwards, the controller provides active torque, similar to concentric muscle power, during the stance phase when the knee is extending. The motion is constrained to the sagittal plane, that is, flexion and extension. The design of the actuators in the prosthesis is based on healthy human joint pseudo-stiffnesses during preferred walking on level ground. The control of the device makes use of the CLs wearable sensory apparatus, comprising inertial measurements units placed on legs and pelvis, and force-sensitive insoles. Gait phases are automatically detected based on these sensors, using thresholds in force and angular position to transition between them (Parri et al., 2017). To avoid false detection of states and possible hazards, the high-level intention detection constrained the device discretely by selecting the correct task to be executed. The CLs-ɣ prosthesis comprises the motor drivers and control electronics in the leg structure and requires an external power source (battery pack) placed on the pelvis. Electronics of the system are custom made boards to control not only the prosthesis, but also act as interface between the force-sensitive insoles that are instrumented in the shoes of both feet and the inertial measurement units that are attached to the trunk and lower limbs (Crea et al., 2014).

Since novel design changes have been made, and more daily activities are being supported by the device, a thorough evaluation process incorporating biomechanical, physiological, psychological, and physical performance parameters is recommended (Ferreira et al., 2007; Sutherland and Schwaber, 2007; Ghillebert et al., 2019). Besides investigating the physical effort when using the prosthetic prototype, cognitive performance is also determined during a dual task (Pauley and Devlin, 2011; Meier et al., 2012; Knaepen et al., 2015; Morgan et al., 2016; Morgan et al., 2018; De Pauw et al., 2019). Performing a cognitive and motoric task simultaneously is interesting since people overperform common walking trials in a laboratory setting, whereas dual task walking better mimic everyday walking and offer a perspective that laboratory measurements cannot show (Urbanek et al., 2018). Dual task paradigms including walking and an attentional task allow to investigate gait pattern alterations and attentional resources used during the motoric task (Krasovsky et al., 2017).

The objective of this study was to thoroughly evaluate the CLs-ɣ prosthesis during simulated daily activities in laboratory conditions. We hypothesized improved physical performance during stair climbing, sit to stand and walking with the CLs-ɣ compared to the current prosthesis given the limited training time with the novel prosthesis. It was also hypothesized that walking with the CLs-ɣ prosthesis would restore a more symmetrical walking pattern (step length and width, stance and swing phases, and heel pressure), and decrease physical effort (metabolic cost, heart rate and rating of perceived exertion) and cognitive load (reaction time) compared to the current prosthesis.

## Materials and Methods

### Recruitment

Nine participants with a transfemoral amputation (*K*-level > 2) were enrolled in the study (Borrenpohl et al., 2016). The recruitment process was performed by the physiotherapist of an orthopedic rehabilitation and research center (VIGO, Wetteren, Belgium), where experiments took place. Participants were not financially compensated but did receive transportation costs. Medicare Functional Classification level was determined by two independent physiotherapists (Jo Ghillebert and Patrick Van de Vaerd) and in case of disagreement a medical doctor was contacted. Study registration was completed at ClinicalTrials.gov under NCT03376919, and approved by the institutional medical ethics committee of UZ Brussel and Vrije Universiteit Brussel (Belgium, B.U.N. 143201732970) and the Federal Agency for Medicines and Health Products (reference number: 80M0725). All participants were provided written and verbal information about the experimental protocol, potential risks and benefits before giving informed consent to participate in the study.

### Experimental Protocol

**Figure 2.**
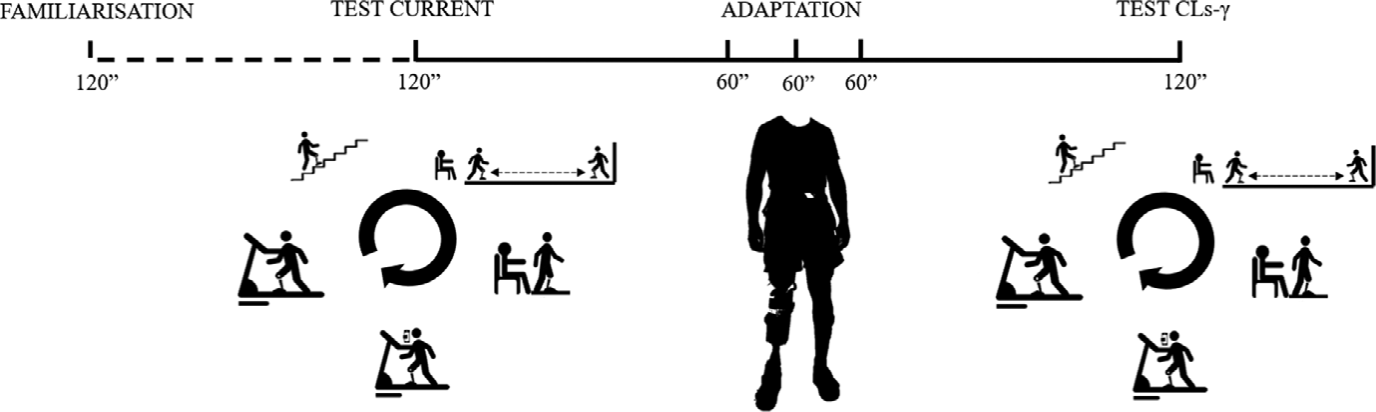
Experimental protocol including a familiarization trial—an experimental trial with the current prosthesis (passive or quasi-passive device)—three adaptation sessions to the novel prosthesis (i.e., the CYBERLEGs-gamma [CLs-γ] prosthesis)—an experimental trial with the CLs-γ prosthesis. The five tasks were a stair climbing test, a timed-up and go test, a sit to stand test, a 2-min dual task and 6-min treadmill walk test.

Each participant visited the laboratory six times: a familiarization session, an experimental session with their current prosthesis, three training sessions with the CLs-γ prosthesis and an experimental session with the CLs-γ prosthesis (Figure 2) (within subject design). All laboratory visits took place in the morning at the same hour and at least 24 hr between each session was planned to counteract fatigue. The total duration of the experiment was 8 months. The environmental humidity, pressure and temperature of the laboratory was 48 ± 6%, 764 ± 8 mmHg and 24.5 ± 1.6°C; respectively.

The familiarization session (120 min) aimed to accustom participants to the experimental protocol, to get used to the measurement devices and to determine the participants’ preferred walking speed. Preferred walking speed was determined on the treadmill through verbal feedback from the participant. The investigator altered the walking speed until the participant confirmed the speed was consistent with the preferred walking speed (Graham et al., 2008). After the familiarization session, the preferred walking speed was used during the experimental treadmill sessions with the current and CLs-γ prosthesis.

The experimental sessions (120 min per session) with the current and CLs-γ prosthesis consisted of five consecutive tasks with 10 min of rest between each task. Participants started with the stair climbing test (Bennell et al., 2011) (SCT; intraclass correlation coefficient [ICC] ascent: 1.00, descent: 0.89 [Highsmith et al., 2017]). They started in front of a staircase (four steps) and were asked to ascend and descend in a safe way as fast as possible. The use of bilateral handrails was allowed when it was difficult to maintain balance. The ascending phase was initiated with the prosthetic side, whereas the first step of the descending phase was performed with the nonprosthetic leg. The second task was a timed-up and go test (Bennell et al., 2011) (TUG; ICC: 0.88 [Resnik and Borgia, 2011]). Participants stood up from their chair (44 cm total height, without arm support and a straight back), walked three meters, turned around, returned to their chair and ended in a seated position. Next, a sit to stand test (Jones et al., 2013) (STS; ICC: 0.84–0.92 [Jones et al., 2013]) was performed. Participants rose as often as possible from their chair in 30 s without support of their hands. Afterwards, a dual task was performed on a treadmill. Participants walked for 2 min on a treadmill at their preferred walking speed while performing the psychomotor vigilance task (Wilkinson and Houghton, 1982) (ICC: 0.82 [Wilkinson and Houghton, 1982]). Participants had to respond by pushing on a button with the index finger of their dominant hand. Earplugs were required during the task to reduce distraction related to sound. The visual stimulus, displayed as a red dot, was visualized on a dark computer screen with a random time interval between 1,000 and 10,000 ms. The interval stimulus-response onset was set at 500 ms and the distance to the screen was approximately 1 m. The experiments ended with a 6-min walk test (Crapo et al., 2002) (6MWT; ICC: 0.94–0.97 [Lin and Bose, 2008]). Participants walked on a treadmill for 6 min at their preferred walking speed. The order of the experimental tasks was determined according to fattigue level from low to high.

Between the experimental sessions with the current and CLs-γ prosthesis, participants underwent three training sessions (60 min per session). The focus of each training session differed: (a) socket fit and walking, (b) alignment and SCT, and (c) STS and TUG. A duplicate of the participants’ current socket was made to optimize fitting and alignment of the novel prosthesis. Participants were fitted and aligned by a certified prosthetist. During each training session, participants also performed hallway walking for 10 min.

### Dependent Measures

Physical performance determinants were gathered in terms of duration (s) of the SCT and TUG, number of cycles during the STS, reaction time (ms) during the psychomotor vigilance task and preferred walking speed (m/s) during the 6MWT. Following spatiotemporal and kinetics data were recorded during walking: cadence (steps/min), maximum heel pressure (N/cm^2^), stance and swing phases (% of gait cycle), step width (cm) and stride length (cm) were reported. Physiological outcome measures were collected in terms of heart rate (bpm) and rating of perceived exertion (score between 0 and 10) after each experimental task. Additionally, oxygen uptake (mL min^−1^ kg^−1^), ventilation (L/min) and metabolic equivalents were gathered during the 6MWT. Psychological outcome measures were determined, such as the visual analogue scale (score on 100) for fatigue and the EuroQol-5D (score on 100) to determine health status (Sung and Wu, 2018; Van Reenen et al., 2018). Other questionnaires were completed during the familiarization session, that is, the prosthetic evaluation questionnaire and the system usability scale (Brooke, 1996; Wurdeman et al., 2018).

### Measurement Devices and Data Analysis

Spatiotemporal and kinetics data were collected with the Zebris software (Medical GmbH, Isny, Germany). Ground reaction force time-series were automatically reduced to zero-dimensional maxima under the forefoot, midfoot and heel. Cadence, stance and swing phases, step width, step and stride length were continuously determined. Gait variability, expressed as the coefficient of variation, was calculated for step width, and step and stride length from the standard deviation dividing by the mean (Guimaraes and Isaacs, 1980). Ergospirometrical data were continuously gathered during the 6MWT using a portable system (Cosmed K5, Cosmed, Rome, Italy). Preceding each test, a calibration (volume, ambient air and reference gas) was performed after a system warm-up period of 30 min. The mixed chamber setting was used, and data was continuously transferred to the Omnia program (Cosmed, Rome, Italy). The device was mounted on the back of the participant with a harness. The net metabolic cost of transport (mL m^−1^ kg^−1^) was calculated from dividing the relative oxygen uptake (mL min^−1^ kg^−1^) by the product of the duration of the test (min), the weight (kg) of the participant and the distance (m) covered during the test (Workman and Armstrong, 2017). Heart rate was measured at the end of each task with an elastic belt strapped around the chest (Polar M400, H7-sensor, Kempele, Finland). The performance outcome measure of the psychomotor vigilance task was reaction times, measured with E-prime 3.0 (Psychology Software Tools, Sharpsburg, MD).

### Statistical Analysis

Data are presented as mean ± standard deviation. SPSS version 25.0 (IBM, New York, NY) was used for statistical analyses. Shapiro Wilk normality tests showed that most datasets were not normally distributed. Therefore, nonparametric Wilcoxon-signed rank tests were conducted. The critical alpha for all analyses was set at 0.05. Hedges’ effect sizes were calculated from dividing the absolute standard test statistics by the square root of the number of observations (Rosenthal, 1994). Small, medium and large effects were considered as 0.2, 0.5, and 0.8, respectively.

## Results

One participant withdrew after the first session for reasons not related to the study. Therefore, data analysis was performed on eight participants (age: 55 ± 15 years; height: 174 ± 5 cm; weight: 81 ± 11 kg; Table 1). An equal number of participants had an amputation of the right or left lower limb. Four participants’ current prosthesis was passive, the others wore a quasi-passive device (microprocessor-controlled). Years since amputation varied among participants (22 ± 14 years), but they were all familiar with their current prosthesis for a minimum duration of 3 months.

**Table 1.** Participants’ characteristics: demographic data, prosthetic components, and walking speed

Participant (gender)	Age (years)	Body weight (kg)	Body length (cm)	AMP side	Length stump (cm)	Suspension mechanism	Current prosthetic knee and ankle joint	Years since amputation	Speed (m/s) current	Speed (m/s) CLs-γ
1 (M)	65	72	172	L	28	Vacuum	Össur Total knee Össur Flex walk	41	0.83	0.33
2 (M)	34	76	178	R	36	Liner	Otto Bock C-leg Otto Bock Taleo	9	1.25	0.56
3 (M)	35	75	176	L	35	Vacuum	Össur Total Knee Össur Variflex	19	0.88	0.56
4 (M)	72	90	172	L	38	Strap	Otto Bock Genium Otto Bock Taleo	7	0.83	0.33
5 (M)	63	98	178	R	28	Vacuum	Össur Mauch Össur Variflex	29	0.44	0.33
6 (F)	55	83	165	R	30	Strap	Össur Total knee Össur Flex walk	34	0.61	0.33
7 (M)	56	90	179	L	35	Vacuum	Otto Bock C-leg Otto Bock C-walk	5	0.56	0.56
8 (M)	61	63	170	R	35	Liner	Otto Bock C-leg Otto Bock C-walk	30	0.83	0.56

*Note:* All participants were indicated as Medicare Functional Classification Level K3 ambulators and had their current prosthesis for at least 3 months.

Abbreviations: AMP, amputated; CLs-γ, CYBERLEGs-gamma; F, female; L, left; M, male; SACH, solid ankle cushion heel.

### Biomechanical Outcomes

**Figure 3.**
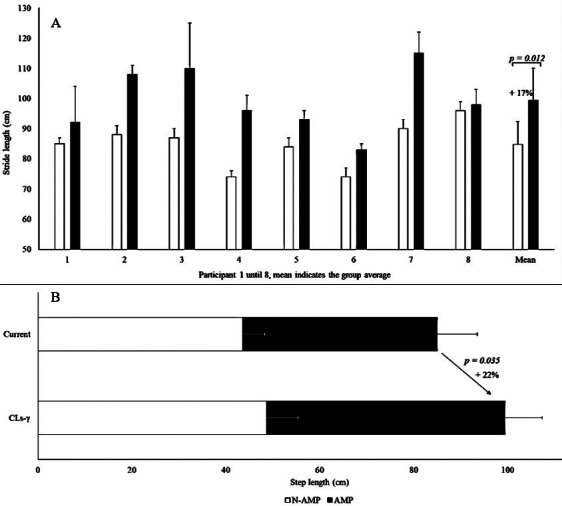
Mean and standard deviation for stride (a) and step length (b) are presented while walking with the current compared to the CYBERLEGs-gamma (CLs-γ) prosthesis. AMP, amputated leg; N-AMP, non-amputated leg.

Stride length significantly increased when walking with the CLs-γ compared to the current prosthesis (17 ± 10%, *p* = .012; Table 2 and Figure 3a). The increased stride length was due to a greater step length of the prosthetic leg (22 ± 20%, *p* = .035; Table 2 and Figure 3b). No significant difference in step length of the nonprosthetic leg was reported (Figure 3b) as well as for step width and gait phases. Additionally, coefficients of variation did not differ between both prostheses for stride length, step width, and step length of the amputated and nonprosthetic leg. Maximum heel pressure of the amputated and nonprosthetic leg did not change while walking with the current compared to the CLs-γ prosthesis.

**Table 2.** Dependent variables are presented as mean and standard deviation with the current and CYBERLEGs-gamma prosthesis, their corresponding *p*-value, absolute standard test value *Z* and effect size

Variables	Current	CLs-γ	*p*-value	Z	Effect size	Variables	Current	CLs-γ	*p*-value	Z	Effect size
Stride length (cm)	84.75 (7.57)	99.38 (10.74)	**.012**	2.52	0.63	Ventilation (L/min)	31.46 (7.83)	29.61 (7.33)	.398	0.85	0.28
Step length AMP (cm)	63.50 (0.50)	75.88 (4.20)	**.035**	2.10	0.53	Metabolic equivalents	4.15 (1.13)	3.86 (1.20)	.271	1.10	0.28
Step length N-AMP (cm)	43.63 (0.70)	48.75 (4.00)	.231	1.26	0.32	Netto metabolic cost (mL m^−1^ kg^−1^)	0.35 (0.13)	0.56 (0.16)	**.028**	2.97	0.74
Step width (cm)	16.13 (5.00)	15.75 (3.06)	.733	0.34	0.09	RPE SCT	2.00 (0.76)	2.50 (1.07)	.334	0.97	0.42
CV stride length	0.06	0.03	.075	1.78	0.45	RPE TUG	1.90 (1.00)	1.75 (0.46)	.739	0.33	0.08
CV step width	0.13	0.09	.235	1.19	0.30	RPE STS	3.00 (0.93)	2.63 (1.19)	.180	1.34	0.34
CV step length AMP	0.07	0.06	.443	0.77	0.19	RPE 6MWT	3.25 (1.04)	3.38 (1.30)	.480	0.71	0.18
CV step length N-AMP	0.09	0.05	.674	0.41	0.11	VAS SCT	2.09 (1.41)	3.48 (2.00)	.093	1.68	0.42
Stance phase AMP (%)	63.71 (3.19)	60.81 (9.00)	.327	0.98	0.25	VAS TUG	2.00 (2.02)	2.03 (1.90)	.672	0.42	0.11
Stance phase N-AMP (%)	73.76 (4.08)	76.88 (7.74)	.263	1.12	0.28	VAS STS	3.73 (2.15)	3.34 (2.08)	.553	0.57	0.15
Swing phase AMP (%)	35.26 (5.16)	34.89 (10.50)	.327	0.98	0.25	VAS 6MWT	3.48 (3.04)	3.63 (2.36)	.735	0.34	0.08
Swing phase N-AMP (%)	27.26 (2.04)	27.43 (10.10)	.207	1.26	0.32	EQ-5D	84.38 (9.43)	87.89 (9.05)	.059	1.89	0.47
Heel pressure AMP (N cm^2^)	18.99 (4.75)	24.19 (5.41)	.063	1.86	0.46	Speed (m/s)	0.77 (0.25)	0.45 (0.12)	**.018**	2.37	0.59
Heel pressure N-AMP (N cm^2^)	19.68 (6.17)	22.04 (3.91)	.575	0.56	0.14	Cadence (steps/min)	71 (13)	60 (9)	**.012**	2.52	0.63
Heart rate SCT (bpm)	102 (18)	106 (17)	.611	0.51	0.13	Reaction time (m)	337 (56)	398 (65)	**.012**	2.52	0.63
Heart rate TUG (bpm)	100 (16)	100 (17)	.533	0.59	0.15	Duration SCT (s)	16 (8)	36 (13)	**.012**	2.52	0.63
Heart rate STS (bpm)	108 (18)	104 (17)	.204	1.27	0.32	Duration TUG (s)	15 (6)	20 (6)	**.012**	2.52	0.63
Heart rate 6MWT (bpm)	120 (25)	113 (18)	.893	0.41	0.10	Number of stands	8.00 (3.00)	7.80 (3.28)	.671	0.43	0.11
Oxygen consumption (mL min^−1^ kg^−1^)	14.15 (4.01)	14.10 (3.80)	.499	0.68	0.17						

Abbreviations: AMP, prosthetic leg; CLs-γ, CYBERLEGs-gamma; CV, coefficient of variation; EQ-5D, EuroQol-5D; N-AMP, nonprosthetic leg; RPE, rating of perceived exertion; SCT, stair climbing test; STS, sit to stand test; TUG, timed-up and go test; VAS, visual analogue scale; 6MWT, 6-min walk test.

Bold entries are significant (<0.05).

### Physiological Outcomes

**Figure 4.**
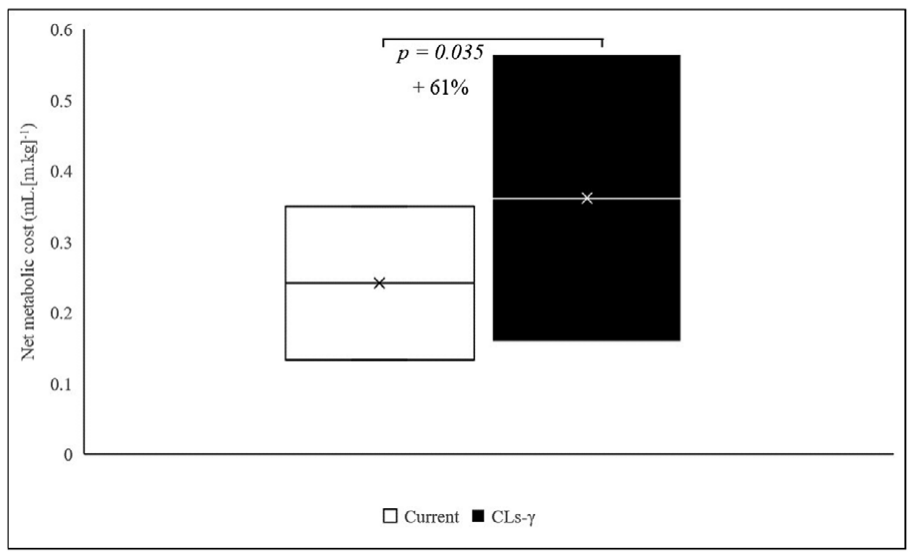
Representation of the net metabolic cost per meter during the 6-min treadmill walk test with the current compared to the CYBERLEGs-gamma (CLs-γ) prosthesis.

Heart rate at the end of each task did not change when wearing the current compared to the CLs-γ prosthesis. The amount of oxygen consumption, ventilation and metabolic equivalents did not vary between walking with both prostheses. However, the net metabolic cost of transport significantly increased when wearing the CLs-γ compared to the current prosthesis during the 6MWT (*p* = .028; Table 2 and Figure 4).

### Psychological Outcomes

No differences in rating of perceived exertion and the visual analogue scale for fatigue were reported during the different tests between the current and CLs-γ prosthesis. The self-reported scores on the EuroQol-5D did not significantly change between both prostheses.

### Physical Performance Outcomes

**Figure 5.**
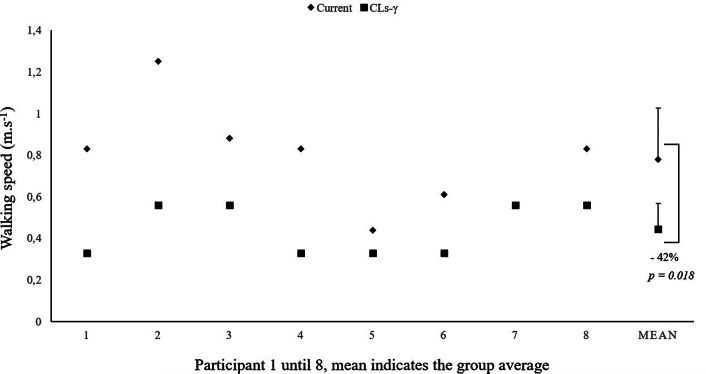
Individual (Williams et al., 2006; Highsmith et al., 2011; Eberly et al., 2014; Simon et al., 2014; Lura et al., 2015; Ledoux et al., 2017; Esposito et al., 2018; Wurdeman et al., 2018) and group average (MEAN) walking speed (mean and standard deviation) with the current (◊) vs. the CYBERLEGs-gamma (CLs-γ) (□) prosthesis are displayed.

Figure 5 shows that the preferred walking speed was significantly lower with the CLs-γ compared to the current prosthesis (*p* = .018; Table 2). Cadence significantly decreased while walking with the CLs-γ compared to the current prosthesis (*p* = .012; Table 2). Furthermore, participants needed significantly more time to respond to the stimulus of the psychomotor vigilance task while walking with the CLs-γ compared to the current prosthesis (*p* = .012; Table 2). Participants also needed significantly more time to complete the SCT and TUG with the CLs-γ compared to the current prosthesis (*p* = .012 and *p* = .012, respectively; Table 2). Number of stands during the STS did not differ between both prostheses.

## Discussion

This study thoroughly evaluated a novel prosthetic prototype during simulated daily activities in individuals with a transfemoral amputation in order to provide an overview on the global functioning of the device as well as more specific information for the next design iteration. The main finding was that the novel motorized prosthesis failed to enhance walking symmetry, physical effort and attentional demand during simulated daily activities compared to current devices. Weight dependent tasks (SCT, TUG, and the 6MWT) were performed worser with the CLs-γ prosthesis compared to the current devices except the increase in stride length during the 6MWT and the way of negotiating stairs. The motorized knee and ankle joint enabled participants to climb the stairs step-over-step, which is impossible with the currently used prosthetic devices where a step-by-step strategy is opted. The weight of a prosthetic device has always been a challenge and especially for motorized two-joint prostheses. Therefore, the recommendation for the next design iteration is to eliminate the weight (current weight of the ankle and knee joint is 2.3 and 3.0 kg, respectively) by selecting carbon-fiber composites and downsizing actuators.

People with a transfemoral amputation have a shorter stride length when walking with their current prosthesis compared to able-bodied individuals, because of the increased moment of inertia of the prosthetic limb during swing phase in typical prostheses (Uchytil et al., 2014). The CLs-γ prosthesis actively flexes during the swing phase, consequently the lever arm decreases between the hip joint (center of rotation) and the center of mass of the prosthesis. Considering the total energy input of the gait cycle remains the same between the current and CLs-γ prosthesis, hence causes an increased angular velocity (Fowles and Cassiday, 2000). The greater angular velocity explains the greater stride length. Although stride length significantly increased with the novel prosthesis, design adaptations are still needed to match able-bodied stride lengths (CLs-γ: 99 ± 11 cm vs. able-bodied: 157 ± 5 cm) (Mahon et al., 2017). Stride length mainly increased as a result from a larger step length of the amputated leg. Though, this exacerbated the asymmetrical walking pattern and did not contribute to a higher symmetry between the amputated and non-amputated leg. Gait variability of stride length, a measure for gait symmetry, confirmed this result as it did not significantly differ between the CLs-γ and current prosthesis, and thus an asymmetrical walking pattern remains present (Guimaraes and Isaacs, 1980).

From a device evaluation perspective this study shows that experimental set-ups can be designed in such a way that multidisciplinary data can be gathered to provide a broad overview of the functioning of the device and the human–prosthetic interaction. The included dual-task paradigm provided a comprehensible insight in how the novel prosthesis negatively influenced the motoric task and attentional demand during both the cognitive and motoric task which is in line with previous findings (Pauley and Devlin, 2011; Meier et al., 2012; Knaepen et al., 2015; Morgan et al., 2016; Morgan et al., 2018; De Pauw et al., 2019). Decreased task and cognitive performance demonstrate task interference, since both walking and the cognitive task require signal processing in similar brain areas (Nijboer et al., 2014). The participants experienced an adaptation, which increased the neural load of wearing the novel prosthesis that is more important than performing an automated pattern such as walking. Theories of central processing assume that attention has a fixed capacity of processing information and that interferences occur at the stimulus identification stage in the occipital lobe (Norman, 1969). Since stimulus processing is limited, a conservative walking pattern is adopted, consisting of a reduced walking speed and wider step width (Morgan et al., 2016). This conservative walking pattern led to reduced postural control demands and minimizes cognitive resources (Morgan et al., 2018). Furthermore, a meta-analysis showed that prosthetic training and rehabilitation can reduce brain information processing demands because of sensorimotor adaptations (Ghai et al., 2017).

Besides design recommendations focused on mechanics, electronics might also be improved to provide necessary sensory information. Advancements have been made in the direction of human-in-the-loop optimization strategies where controller parameters could benefit from biological measures such as the metabolic cost (Handford and Srinivasan, 2016). Passive and active loops, and torque-angle and torque-time optimization could benefit movement efficiency (Handford and Srinivasan, 2018). For example, implementing a push-off control strategy in a motorized ankle prosthesis has shown to improve gait symmetry and reduce the metabolic cost with 14 ± 8% (Feng and Wang, 2017). Therefore, improving performance of the device goes hand in hand with further developments of control algorithms next to the examination in testbeds or emulators (Caputo and Collins, 2014). This highlights the need to integrate more sophisticated control systems taking into consideration individual responses when using a robotic device.

Until now, it remains a challenge for two-joint synchronized devices with impedance or kinematic control-based mechanisms to improve daily functioning. Although high expectations toward improved performance are present, numerous papers reported that motorized devices fail to meet expectations, and thus still need to be further developed (Sup et al., 2009; Simon et al., 2013, 2014; Ingraham et al., 2016). Thus, the thorough examination of the CLs-γ prosthesis also demonstrated premature for market implementation. Though some improvements have been noticed, especially during stair ascending and descending where participants were again able to walk the stairs step-over-step. This reciprocal stair ambulation shows the possible functional capacity of motorized lower limb prostheses. The CLs-γ prosthesis was able to restore stair climbing due to design approximating pseudo-stiffness of the non-amputated joints, which determined the stiffness and bandwidth of the artificial joints. The CLs-γ prosthesis differs from other prototypes since the finite state controller relies on pressure-sensitive insoles in the shoes and inertial measurement units attached to the trunk and lower limbs.

The choice of 3 hr of training with the CLs-γ prosthesis was based on a study that reported clinically relevant outcomes for further development of a prototype after a few hours of training with a powered ankle and knee prosthesis (Simon et al., 2014). However, this limits the results to short-term use of a novel device. To determine long-term adaptations, a training period of 3 months with a device of at least a technology readiness level 6, which refers to a model or prototype that can be demonstrated in a relevant environment, is advised (Assistant Secretary of Defense, 2011). Another limitation of this study is with regards to the study design. Future research should consider exploring the influence of years of amputation on outcome measures during an evaluation procedure. A randomized controlled study design is encouraged going forward.

To conclude, a multidisciplinary approach was adopted to evaluate a novel motorized lower limb prosthetic device. Increased physical effort and cognitive load were required during walking with the CLs-ɣ prosthesis compared to the current prosthesis. All participants were able to conduct stairs with the step-over-step instead of the step-by-step strategy.

## Data Availability

Data content sharing is applicable via special request for data sharing. Please contact the corresponding author.

## Notations

AMP: amputated
CLs-ɣ: CYBERLEGs-gamma
CV: coefficient of variation
EQ-5D: EuroQol-5D
F: female
L: left
M: male
N-AMP: non-amputated leg
R: right
RPE: rating of perceived exertion
SCT: stair climbing test
STS: sit to stand test
TFA: transfemoral amputation
TUG: timed-up and go test
VAS: visual analogue scale
6MWT: six-min treadmill walk test

## References

[r1] Assistant Secretary of Defense (2011) Technology Readiness Assessment (TRA) Guidance.

[r2] Bennell K, Dobson F and Hinman R (2011) Measures of physical performance assessments: Self-Paced Walk Test (SPWT), Stair Climb Test (SCT), Six-Minute Walk Test (6MWT), Chair Stand Test (CST), Timed Up & Go (TUG), Sock Test, Lift and Carry Test (LCT), and Car Task. Arthritis Care & Research 63(SUPPL. 11), 350–370. 10.1002/acr.20538 22588756

[r3] Borrenpohl D, Kaluf B and Major MJ (2016) Survey of U.S. practitioners on the validity of the medicare functional classification level system and utility of clinical outcome measures for aiding K-level assignment. Archives of Physical Medicine and Rehabilitation 97(7), 1053–1063. 27016261 10.1016/j.apmr.2016.02.024

[r4] Brooke J (1996) SUS: A quick and dirty usability scale industrial usability evaluation. In Usability Evaluation in Industry, vol. 189, no. 194, pp. 4–7.

[r5] Caputo JM and Collins SH (2014) A universal ankle-foot prosthesis emulator for human locomotion experiments. Journal of Biomechanical Engineering 136(3), 1–10. 10.1115/1.4026225 24337103

[r6] Crapo RO, Enright PL and Zeballos RJ (2002) ATS statement: The six-minute walk test. American Journal of Respiratory and Critical Care Medicine 166, 111–117. 12091180 10.1164/ajrccm.166.1.at1102

[r7] Crea S, Donati M, De Rossi SMM, Maria Oddo C and Vitiello N (2014) A wireless flexible sensorized insole for gait analysis. Sensors 14(1), 1073–1093. 24412902 10.3390/s140101073 PMC3926603

[r8] De Pauw K, Cherelle P, Tassignon B, Van Cutsem J, Roelands B, Marulanda FG, Lefeber D, Vanderborght B and Meeusen R (2019) Cognitive performance and brain dynamics during walking with a novel bionic foot: A pilot study. PLoS One 14(4), 1–17. 10.1371/journal.pone.0214711 PMC6447229 30943265

[r9] Eberly VJ, Mulroy SJ, Gronley JK, Perry J, Yule WJ and Burnfield JM (2014) Impact of a stance phase microprocessor-controlled knee prosthesis on level walking in lower functioning individuals with a transfemoral amputation. Prosthetics and Orthotics International 38(6), 447–455. 24135259 10.1177/0309364613506912

[r10] Esposito ER, Rábago CA and Wilken J (2018) The influence of traumatic transfemoral amputation on metabolic cost across walking speeds. Prosthetics and Orthotics International 42(2), 214–222. 28655287 10.1177/0309364617708649

[r11] Feng Y and Wang Q (2017) Combining push-off power and nonlinear damping behaviors for a lightweight motor-driven transtibial prosthesis. IEEE/ASME Transactions on Mechatronics 22(6), 2512–2523.

[r12] Ferreira J, Noble J and Biddle R (2007) Agile development iterations and UI design. In Proceedings of Agile Development Conference (AGILE 2007), September, pp. 50–58.

[r13] Flynn L, Geeroms J, Jimenez-Fabian R, Vanderborght B, Vitiello N and Lefeber D (2015) Ankle–knee prosthesis with active ankle and energy transfer: Development of the CYBERLEGs Alpha-Prosthesis. Robotics and Autonomous Systems 73, 4–15. Accessed date 13 may 2020. Available at https://www.sciencedirect.com/science/article/pii/S0921889014003108.

[r14] Flynn LL, Geeroms J, Van Der Hoeven T, Vanderborght B and Lefeber D (2018) VUB-CYBERLEGs CYBATHLON 2016 Beta-Prosthesis: Case study in control of an active two degree of freedom transfemoral prosthesis. Journal of Neuroengineering and Rehabilitation 15(1), 1–16. 29298695 10.1186/s12984-017-0342-y PMC5751827

[r15] Fowles GR and Cassiday GL (2000) Anaytical mechanics. American Journal of Physics 68(4), 390–394.

[r16] Geeroms J, Flynn L, Jimenez-Fabian R, Vanderborght B, Lefber D (2017) Design and energetic evaluation of a prosthetic knee joint actuator with a lockable parallel spring. Bioinspiration and Biomimetics 12(3), 212–220. 10.1088/1748-3190/aa575c 28059775

[r17] Ghai S, Ghai I and Effenberg AO (2017) Effects of dual tasks and dual-task training on postural stability: A systematic review and meta-analysis. Clinical Interventions in Aging 12, 557–577. 28356727 10.2147/CIA.S125201 PMC5367902

[r18] Ghillebert J, De Bock S, Flynn L, Geeroms J, Tassignon B, Roelands B, Lefeber D, Vanderborght B, Meeusen R and De Pauw K (2019) Guidelines and recommendations to investigate the efficacy of a lower-limb prosthetic device: A systematic review. IEEE Transactions on Medical Robotics and Bionics 1(4), 279–296.

[r19] Graham JE, Ostir GV, Fisher SR and Ottenbacher KJ (2008) Assessing walking speed in clinical research: A systematic review. Journal of Evaluation in Clinical Practice 14(4), 552–562. 18462283 10.1111/j.1365-2753.2007.00917.x PMC2628962

[r20] Guimaraes RM and Isaacs B (1980) Characteristics of the gait in old people who fall. International Rehabilitation Medicine 2(4), 177–180. 7239777 10.3109/09638288009163984

[r21] Handford ML and Srinivasan M (2016) Robotic lower limb prosthesis design through simultaneous computer optimizations of human and prosthesis costs. Scientific Reports 6, 19983. 26857747 10.1038/srep19983 PMC4746571

[r22] Handford ML and Srinivasan M (2018) Energy-optimal human walking with feedback-controlled robotic prostheses: A computational study. IEEE Transactions on Neural Systems and Rehabilitation Engineering 26(9), 1773–1782. 30040647 10.1109/TNSRE.2018.2858204

[r23] Highsmith MJ, Kahle JT, Carey SL, Lura DJ, Dubey RV, Csavina KR and Quillen WS (2011) Kinetic asymmetry in transfemoral amputees while performing sit to stand and stand to sit movements. Gait & Posture 34(1), 86–91. 21524913 10.1016/j.gaitpost.2011.03.018

[r24] Highsmith MJ, Kahle JT, Kaluf B, Miro RM, Mengelkoch LJ and Klenow TD (2017) Psychometric evaluation of the hill assessment index (HAI) and stair assessment index (SAI) in high-functioning transfemomral amputees. Technology and Innovation 18(813), 193–201. 10.21300/18.2-3.2016.193 PMC5218524 28066528

[r25] Ingraham KM, Fey NP, Simon AM and Hargrove LJ (2016) Assessing the relative contributions of active ankle and knee assistance to the walking mechanics of transfemoral amputees using a powered prosthesis. PLoS One 11(1), 1–19. 10.1371/journal.pone.0147661 PMC4725744 26807889

[r26] Jones CJ, Rikli RE and Beam WC (2013) A 30-s chair-stand test as a measure of lower body strength in community-residing older adults. Research Quarterly for Exercise and Sport 70(2), 113–119. 10.1080/02701367.1999.10608028 10380242

[r27] Knaepen K, Marusic U, Crea S, Rodríguez Guerrero CD, Vitiello N, Pattyn N, Mairesse O, Lefeber D and Meeusen R (2015) Psychophysiological response to cognitive workload during symmetrical, asymmetrical and dual-task walking. Human Movement Science 40, 248–263. 25617994 10.1016/j.humov.2015.01.001

[r28] Krasovsky T, Weiss PL and Kizony R (2017) A narrative review of texting as a visually-dependent cognitive-motor secondary task during locomotion. Gait & Posture 52, 354–362. 28043057 10.1016/j.gaitpost.2016.12.027

[r29] Lechler K, Frossard B, Whelan L, Langlois D, Müller R and Kristjansson K (2018) Motorized biomechatronic upper and lower limb prostheses - Clinically relevant outcomes. The American Academy of Physical Medicine and Rehabilitation 10(9), S207–S219. 10.1016/j.pmrj.2018.06.015 30269806

[r30] Ledoux ED, Member S and Goldfarb M (2017) Control and evaluation of a powered transfemoral prosthesis for stair ascent. IEEE Transactions on Neural Systems and Rehabilitation Engineering 4320, 1–8. 10.1109/TNSRE.2017.2656467 28113346

[r31] Lin SJ and Bose NH (2008) Six-minute walk test in persons with transtibial amputation. Archives of Physical Medicine and Rehabilitation 89(12), 2354–2359. 18976979 10.1016/j.apmr.2008.05.021

[r32] Lura DJ, Wernke MM, Carey SL, Kahle JT, Miro RM and Highsmith MJ (2015) Clinical biomechanics differences in knee flexion between the Genium and C-Leg microprocessor knees while walking on level ground and ramps. Clinical Biomechanics 30, 175–181. 25537443 10.1016/j.clinbiomech.2014.12.003

[r33] Mahon CE, Pruziner AL, Hendershot BD, Wolf EJ, Darter BJ, Foreman KB and Webster JB (2017) Gait and functional outcomes for young, active males with traumatic unilateral transfemoral limb loss. Military Medicine 182(7), 1913–1923. 10.7205/MILMED-D-16-00356 28810990

[r34] Martinez-Villalpando EC, Mooney L, Elliott G and Herr H (2011) Antagonistic active knee prosthesis. A metabolic cost of walking comparison with a variable-damping prosthetic knee. Annual International Conference of the IEEE Engineering in Medicine and Biology Society 2011, 8519–8522. 22256326 10.1109/IEMBS.2011.6092102

[r35] Meier M, Hansen A, Gard SA and Mcfadyen A (2012) Obstacle course: Users’ maneuverability and movement efficiency when knee joints. Journal of Rehabilitation Research and Development 49(4), 583–596. 22773261 10.1682/jrrd.2010.05.0094

[r36] Morgan SJ, Hafner BJ, Kartin D and Kelly VE (2018) Dual-task standing and walking in people with lower limb amputation: A structured review. Prosthetics and Orthotics International 42(6), 652–666. 30047839 10.1177/0309364618785728

[r37] Morgan SJ, Hafner BJ and Kelly VE (2016) The effects of a concurrent task on walking in persons with transfemoral amputation compared to persons without limb loss. Prosthetics and Orthotics International 40(4), 490–496. 26209423 10.1177/0309364615596066

[r38] Nijboer M, Borst J, van Rijn H and Taatgen N (2014) Single-task fMRI overlap predicts concurrent multitasking interference. NeuroImage 100, 60–74. 24911376 10.1016/j.neuroimage.2014.05.082

[r39] Norman DA (1969) Memory while shadowing. Quarterly Journal of Experimental Psychology 21, 85–93. 5777987 10.1080/14640746908400200

[r40] Parri A, Martini E, Geeroms J, Flynn L, Pasquini G, Crea S, Lova RM, Lefeber D, Kamnik R, Munih M and Vitiello N (2017) Whole body awareness for controlling a robotic transfemoral prosthesis. Frontiers in Neurorobotics 11, 1–14. 28611621 10.3389/fnbot.2017.00025 PMC5448151

[r41] Pauley T and Devlin M (2011) Influence of a concurrent cognitive task on foot pedal reaction time following traumatic, unilateral transtibial amputation. Journal of Rehabilitation Medicine 43(11), 1020–1026. 22031348 10.2340/16501977-0880

[r42] Resnik L, Borgia M (2011) Reliability of outcome measures for people with lower-limb amputations: Distinguishing true change from statistical error. Physical Therapy 91(4), 555–565. 21310896 10.2522/ptj.20100287

[r43] Rosenthal R (1994) Parametric measures of effect size. In Cooper H and Hedges LV (eds), The Handbook of Research Synthesis. New York, pp. 231–244.

[r44] Simon AM, Fey NP, Finucane SB, Lipschutz RD and Hargrove LJ (2013) Strategies to reduce the configuration time for a powered knee and ankle prosthesis across multiple ambulation modes. IEEE International Conference on Rehabilitation Robotics 2013, 1–6. 10.1109/ICORR.2013.6650371 24187190

[r45] Simon AM, Ingraham KA, Fey NP, Finucane SB, Lipschutz RD, Young AJ and Hargrove LJ (2014) Configuring a powered knee and ankle prosthesis for transfemoral amputees within five specific ambulation modes. PLoS One 9(6), 1–10. 10.1371/journal.pone.0099387 PMC4051756 24914674

[r46] Sung YT and Wu JS (2018) The visual analogue scale for rating, ranking and paired-comparison (VAS-RRP): A new technique for psychological measurement. Behavior Research Methods 50(4), 1694–1715. 29667082 10.3758/s13428-018-1041-8 PMC6096654

[r47] Sup F, Varol HA, Mitchell J, Withrow TJ and Goldfarb M (2009) Self-contained powered knee and ankle prosthesis: Initial evaluation on a transfemoral amputee. IEEE International Conference on Rehabilitation Robotics 2009, 638–644. 20228944 10.1109/ICORR.2009.5209625 PMC2836171

[r48] Sutherland J and Schwaber K (2007) The Scrum Papers: Nuts, Bolts, and Origins of an Agile Process.

[r49] Takahashi KZ, Horne JR and Stanhope SJ (2015) Comparison of mechanical energy profiles of passive and active below-knee prostheses: A case study. Prosthetics and Orthotics International 39(2), 150–156. 24418933 10.1177/0309364613513298

[r50] Uchytil J, Jandacka D, Zahradnik D, Farana R and Janura M (2014) Temporal-spatial parameters of gait in transfemoral amputees: Comparison of bionic and mechanically passive knee joints. Prosthetics and Orthotics International 38(3), 199–203. 23824546 10.1177/0309364613492789

[r51] Urbanek JK, Zipunnikov V, Harris T, Crainiceanu C, Harezlak J and Glynn NW (2018) Validation of gait characteristics extracted from raw accelerometry during walking against measures of physical function, mobility, fatigability, and fitness. The Journals of Gerontology. Series A, Biological Sciences and Medical Sciences 73(5), 676–681. 28958000 10.1093/gerona/glx174 PMC5905654

[r52] Van Reenen M, Oppe M, Boye K, Herdman M, Kennedy-Martin M, Kenndy-Martin T, Slaap B (2018) EuroQol Research Foundation. EQ-5D-3L User Guide. EuroQol Research Foundation, pp. 7–8.

[r53] Wilkinson RT and Houghton D (1982) Field test of arousal: A portable reaction timer with data storage. Human Factors 24(4), 487–493. 7129455 10.1177/001872088202400409

[r54] Williams RM, Turner AP, Orendurff M, Segal AD, Klute GK, Pecoraro J and Czerniecki J (2006) Does having a computerized prosthetic knee influence cognitive performance during amputee walking? Archives of Physical Medicine and Rehabilitation 87(7), 989–994. 16813788 10.1016/j.apmr.2006.03.006

[r55] Workman JM and Armstrong BW (2017) Metabolic cost of walking: equation and model. Journal of Applied Physiology 61(4), 1369–1374. 10.1152/jappl.1986.61.4.1369 3781952

[r56] Wu M, Haque MR and Shen X (2017) Obtaining natural sit-to-stand motion with a biomimetic controller for powered knee prostheses. Journal of Healthcare Engineering 2017, 3850351. 29075428 10.1155/2017/3850351 PMC5624172

[r57] Wurdeman SR, Stevens PM and Campbell JH (2018). Mobility analysis of amputees (MAAT I): Quality of life and satisfaction are strongly related to mobility for patients with a lower limb prosthesis. Prosthetics and Orthotics International 42(5), 498–503. 28990467 10.1177/0309364617736089 PMC6146310

